# Investigating the association of physical and psychological problems with the levels of interleukin-1 and -6 in COVID-19 patients

**DOI:** 10.3389/fpsyt.2023.1241190

**Published:** 2023-08-25

**Authors:** Galia Bahadori-Birgani, Shahram Molavynejad, Mahbobe Rashidi, Fereshteh Amiri, Elham Maraghi, Bahman Dashtbozorgi, Zulvikar Syambani Ulhaq, Gholamreza Alizadeh-Attar

**Affiliations:** ^1^Nursing Care Research Center in Chronic Diseases, School of Nursing and Midwifery, Ahvaz Jundishapur University of Medical Science, Ahvaz, Iran; ^2^Pain Research Center, Ahvaz Jundishapur University of Medical Science, Ahvaz, Iran; ^3^Department of Epidemiology and Biostatistics, School of Health, Ahvaz Jundishapur University of Medical Sciences, Ahvaz, Iran; ^4^Research Center for Pre-Clinical and Clinical Medicine, National Research and Innovation Agency Republic of Indonesia, Cibinong, Indonesia; ^5^School of Medicine, Ahvaz Jundishapur University of Medical Sciences, Ahvaz, Iran

**Keywords:** interleukin 1, interleukin 6, inflammatory mediator, COVID-19, depression, anxiety, stress, physiological indicators

## Abstract

**Introduction:**

The COVID-19 virus spreads at a high rate, exerting many physical, mental and psychological effects on patients. Patients with COVID-19 have been reported to have high levels of interleukin 1 and interleukin 6. Therefore, this study was conducted to determine the association of physical, mental, and psychological problems with the levels of interleukin-1 and -6 in COVID-19 patients.

**Methodology:**

This is cross-sectional descriptive-analytical research on 121 COVID-19 patients selected using simple random sampling method. The patients were hospitalized in university hospitals affiliated to Ahvaz Jundishapur University of Medical Sciences and Amir al-Momenin Hospital. Data collection tools included the depression anxiety stress scale (DASS), a demographic questionnaire, and a checklist of physical problems. Blood sampling was also done to perform an ELISA test and measure the level of interleukin-1 and -6. Data were analyzed based on independent *t*-tests, chi-square, regression, and Pearson’s correlation coefficient, using SPSS ver. 22.

**Results:**

The average age of the 121 patients participating in this study was 53.31 ± 14.09. A direct and statistically significant correlation was observed between body temperature on the first day and interleukin 1 level. A statistically significant negative correlation was observed between blood oxygen saturation level and interleukin-1 and -6 on the first, third and fifth days. Shortness of breath and coughing had a statistically significant correlation with the level of interleukin 1 on the third and fifth days. A direct and statistically significant correlation was observed between body temperature on the first, third and fifth days and interleukin 6 level. Coughing on the third and fifth days had a statistically significant relationship with interleukin 6 level. No direct and non-significant statistical correlation was found between depression and stress and the serum level of interleukin 1, but a significant correlation was observed between anxiety and serum level of interleukin 1. Finally, the results showed that depression, anxiety and stress had a direct and statistically significant correlation with the serum level of interleukin 6.

**Conclusion:**

Given the relationship between interleukin-1 and -6 and most physical and psychological problems, level of the inflammatory biomarkers interleukin-1 and -6 can be used to estimate the severity of physical and psychological symptoms in COVID-19 patients.

## Introduction

SARS-CoV-2 (2019-nCoV) coronavirus is the seventh pathogenic virus in humans that can cause severe human disease along with severe acute respiratory syndrome (SARS) (1, 2). Disturbance in the body’s immune system caused by corona virus infection can lead to psychological pathology and psychiatric consequences after the outbreak of acute respiratory syndrome (3). Patients with COVID-19 are really vulnerable to stressful situations, and they have low psychological tolerance. According to the current state of the disease in the world, these individuals are highly prone to psychological disorders such as emotional changes, depression, anxiety, frustration, fear, high level of mental stress, negative thoughts, and insomnia (4, 5). Corona viruses can cause mental complications through direct viral infection of the central nervous system (CNS) or indirectly through the immune response (6). Other factors threatening the mental health of people in the community include fear of illness or death, spread of false news and rumors, disruption in daily activities, prohibition or restrictions on travel, reduced social relations, and job and financial problems, to name only a few (7). One of the most important psychological disorders that can harm the mental health of patients with COVID-19 is post-traumatic stress disorder (PTSD), which may lead to permanent consequences such as an influx of disturbing memories, avoidance behaviors, irritability, and emotional numbness (8). Other important psychological disorders that can negatively affect the mental health of patients with COVID-19 are stress, anxiety and depression. The result of a meta-analysis in patients with COVID-19 showed that the pooled prevalence of depression was 45% (95% CI: 37%–54%, *I*^2^ = 96%), the pooled prevalence of anxiety was 47% (95% CI: 37%–57%, *I*^2^ = 97%), and the pooled prevalence of sleeping disturbances was 34% (95% CI: 19%–50%, *I*^2^ = 98%) (9). The prevalence of depressive and anxiety symptoms, insufficient sleep, unsatisfactory sleep, and unsatisfactory quality of life was significantly increased. Importantly, a significant relation with all mental health outcomes considered was found for women vs. males, for current vs. never smokers, and with increasing physical activity. In addition, the use of at least one psychotropic drug increased compared to pre-lockdown (10–12). Patients infected with COVID-19 show high levels of the following cytokines: IL-1, IL-6, IFN, CXCL10, and CCL2 (13). Activation of immune cells residing in the lung through diagnostic receptors triggers the release of inflammatory cytokines and the release of blood neutrophils and monocytes into the bronchi, which will cause damage to the lung tissue and subsequently destruction of alveolar ventilation (14). Increased levels of interleukin-6 have been proven in cases of inflammatory diseases. COVID-19 is pathophysiologically characterized with severe inflammation and chemokine storm, which is associated with increased levels of interleukin-1 and 6 (15, 16). IL-6 exerts various effects other than those on hepatocytes and lymphocytes and these are frequently detected in chronic inflammatory diseases (17). The results of Zhu’s et al. (18) study showed that high levels of interleukin-6, C-reactive proteins, and high blood pressure were independent risk factors for the severity of COVID-19. Some studies have reported the relationship of mental and psychological disorders with high levels of interleukin-1 and -6 (19–21). Interleukin 6 plays a role in the development and physiological or somatic consequences of major depression, and the levels of two types of cytokines, namely interleukin 6 and tumor necrosis factor α (TNFα), are significantly higher in patients with major depression. In general, cytokine dysfunction could be associated with depression (22). Despite the prevalence of COVID-19 in the world and Iran and the numerous studies conducted so far, little is still known about this disease. In addition to physical symptoms in COVID-19 patients, psychological symptoms among hospitalized patients are of particular importance to nurses since they play a key role in the recovery process and improving the physical and mental state of these patients (23). Currently, available evidence overall argues the COVID-19 in the severe state exhibits a cytokine storm with elevated plasma levels IL-1, and IL-6. However, crucial questions remain unanswered pertaining to the physical and psychological problems with the levels of interleukin-1 and -6 in COVID-19 patients. To address these questions, we aimed to examine the association of physical and psychological problems with the levels of interleukin-1 and -6 in patients infected with COVID-19.

## Materials and methods

### Design

This was a cross-sectional descriptive-analytical research conducted on 121 COVID-19 patients hospitalized in Amir al-Momenin Hospital and university hospitals affiliated to Ahvaz Jundishapur University of Medical Sciences, Ahvaz, Iran.

### Participants

The participants of the present study were selected using convenient sampling from among COVID-19 patients hospitalized in the general wards of Amir al-Momenin Hospital and university hospitals affiliated to Ahvaz Jundishapur University of Medical Sciences.

The sample size was calculated using PASS version 15 based on the Multiple Regression using Effect Size procedure. Assuming age and gender as control variables, interleukin level as the test variable, type 1 error of 0.05, power of 80%, and an effect size of 0.08, the final sample size was calculated to be 121.

Patients were eligible to enter the study if they: were diagnosed with COVID-19 and in the severe stage of the disease (positive PCR test), aged between 18–65 years, were hospitalized in either Amir al-Momenin Hospital or university hospitals affiliated to Ahvaz Jundishapur University of Medical Sciences, and gave informed written consent to participate in the study. Exclusion criteria were: inadequate completion of the questionnaires or unwillingness to continue participation.

### Instruments

The data collection tools used in this study were the depression, anxiety and stress scale (DASS-21) and a demographic questionnaire inquiring information about age, sex, history of neurological and psychiatric diseases, history of taking neuropsychiatric drugs, history of suicide attempts, marital status, employment status, and level of education. Also, a checklist was used to record the values of interleukin-1 and -6 in the patients’ tests. Physical problems (i.e., abnormal body temperature and blood oxygen saturation levels, shortness of breath, coughing, weakness, and lethargy) were also recorded in a separate checklist. Body temperature was measured using a non-contact thermometer (Beurer Company, Germany) and oxygen saturation level was measured using a pulse oximeter (Beurer Company, Germany). Shortness of breath, coughing, weakness, and lethargy were assessed based on examination, observation, and patient report.

Persian version of depression, anxiety, and stress scale is a set of three self-measurement subscales to assess negative affective states of depression, anxiety and stress. Each of the three subscales of DASS-21 contains 7 items (depression; items 3, 5, 10, 13, 16, 17, and 21; anxiety; items 2, 4, 7, 9, 15, 19, and 20; stress; items 1, 6, 8, 11, 12, 14, and 18). The depression scale assesses dysphoria, hopelessness, devaluation of life, self-deprecation, lack of interest/involvement, anhedonia and inertia. The anxiety scale assesses autonomic arousal, skeletal muscle effects, situational anxiety, and subjective experience of anxious affect. The stress scale is sensitive to levels of chronic nonspecific arousal. It assesses difficulty relaxing, nervous arousal, and being easily upset/agitated, irritable/over-reactive and impatient. Scores for depression, anxiety and stress are calculated by summing the scores for the relevant items. Participants were asked to rate each item’s applicability based on their past week experience on a four-point scale ranged from 0 (“does not apply to me at all”) to 3 (“applies to me most of the time”). In each scale, total scores were calculated by summing the scores into seven items and then multiplied by two. Total scores ranged between 0 and 42 (24). Also, the test does not have reverse scores. The original study reported the high reliability of DASS-21, Cronbach’s alpha coefficients for depression, anxiety, and stress were reported as 0.91, 0.84, and 0.90, respectively (25). Numerous studies have evaluated the psychometric properties of this scale in medical and non-medical populations to determine its validity and reliability (26, 27). In Iran, Kakemam et al. (28) prepared the Persian version of DASS and validated it. The internal reliability of DASS scales was calculated using Cronbach’s alpha and the following results were obtained: 0.93 for the depression scale, 0.79 for the anxiety scale, and 0.91 for the stress scale.

On the first day of hospitalization, questionnaires and checklists were completed in full compliance with health precautionary measures during the COVID-19 pandemic. Physical problems were also recorded using the above-mentioned tools. Afterwards, using venous blood sampling, the clot test was taken by staff nurses, and in compliance with the health regulations and standards for maintaining the test tubes, the blood samples were sent to Imam Khomeini Hospital to perform ELISA test and measure the level of interleukin-1 and -6. Finally, the measured values of these two factors were recorded in the checklist of each patient. In the following days until the end of the hospitalization period, physical and mental problems were measured every other day and the patient’s final condition was also recorded. The human IL-6 ELISA kit (Carmania Pars Gene Company, Iran), manufactured according to the standards of Mitrogen and R&D companies, uses an immunoassay method based on sandwich ELISA protocols. In this method, both a special monoclonal antibody against IL-6 and the biotin-streptavidin system are used. This gives the kit very high sensitivity and specificity in measuring interleukin 6. The detection range of this kit is 6.25–200 pg/mL. The main advantages of this method include a sensitivity or detection limit of around 5 pg/mL as well as high specificity and accuracy. This kit can detect natural and human recombinant IL-6. The results were reported in pg/mL (29). The level of IL-β1 was measured in picograms per milliliter based on ELISA method using the quantitative detection kit (Carmania Pars Gene, Iran) manufactured according to the standard of Mitrogen and R&D companies.

### Ethical considerations

This study was approved by the Ethics Committee of Ahvaz Jundishapur University of Medical Sciences (Ref. ID: IR.AJUMS.REC.1400.050). The study was conducted according to the Declaration of Helsinki. Written informed consent was obtained from the patients who were already told that they have the right to withdraw from the research at any stage. The participants and their families had the right to ask any questions to resolve any ambiguity. They were also assured that no cost was imposed on them, that their information would remain confidential, and that the results would be published only for research purposes.

### Data analysis

Chi-square test (or Fisher’s exact test) was used to examine the relationship between qualitative variables, and independent *t*-test and repeated measure analysis was used to compare quantitative variables between two independent groups. Correlation coefficient and multivariate regression models were used to determine the relationship between interleukin level and psychological problems. The significance level of the above tests was set at 0.05. Data analysis was done using SPSS version 22. The normality of the distribution of the variables was checked using the Kolmogorov–Smirnov test.

## Results

This research was conducted on 121 patients with an average age of 53.31 ± 14.09, of whom, 51.4% were women and the majority (52.1%) were unemployed. Other demographic characteristics are listed in Table 1.

**Table 1:** The demographic characteristics of the COVID-19 patients participating in the study.

Quantitative variable	Mean ± SD
Age	53.31 ± 14.09

Qualitative variable	Frequency (percentage)	
Sex	Woman	61 (51.4)
	Man	60 (49.6)
Marital status	Single	2 (1.7)
	Married	96 (79.3)
	Divorced/widow	23 (19)
Occupation	Unemployed	63 (52.1)
	Pensioner	20 (16.5)
	Self-employed	25 (20.7)
	Teacher	3 (2.5)
	Office worker	10 (8.2)
Educational attainment	Illiterate	26 (21.4)
	No high school diploma	41 (33.9)
	High school diploma	25 (20.7)
	University degree	29 (24)
History of neurological disease	Yes	4 (3.3)
	No	117 (96.7)
History of taking neuropsychiatric drugs	Yes	24 (19.8)
	No	97 (80.2)
History of suicide attempts	Yes	2 (1.7)
	No	119 (98.3)

Results of independent *t*-test and chi-square showed that there was no statistically significant difference between male and female patients in terms of marital status, educational attainment, history of neuropsychiatric disease, history of taking neuropsychiatric drugs, and history of suicide attempts (*p* > 0.05). A statistically significant difference was observed between the two groups only in terms of occupation (*p* < 0.0001). Also, regarding interleukin-1 and -6 variables, no statistically significant difference was observed between the two sexes (*p* > 0.05) (Table 2).

**Table 2:** Demographic characteristics of the COVID-19 patients included in the study according to their gender.

Variable	Male (*n* = 60)	Female (*n* = 60)	*p*
Age: mean ± SD	52.61 ± 12.60	54.03 ± 15.53	0.581^a^
Marital status: frequency (%)			0.15^b^
Single	2(3.3)	0 (0)	
Married	44 (73.3)	52 (85.2)	
Divorced/widow	14 (23.3)	9 (14.8)	
Occupation			<0.0001^b^
Unemployed	11 (18.3)	52 (85.2)	
Pensioner	17 (28.3)	3 (4.9)	
Self-employed	23 (38.3)	2 (3.3)	
Teacher	0 (0)	3 (4.9)	
Office worker	9 (15)	1 (1.6)	
Educational attainment			0.12^b^
Illiterate	9 (15)	17 (27.9)	
No high school diploma	26 (43.3)	15 (24.6)	
High school diploma	12 (20)	13 (21.3)	
University degree	13 (21.7)	16 (26.2)	
History of neurological disease			0.61^b^
Yes	1 (1.7)	3 (4.9)	
No	59 (98.3)	58 (95.1)	
History of taking neuropsychiatric drugs			0.65^b^
Yes	13 (21.3)	11 (18)	
No	47 (78.3)	50 (82)	
History of suicide attempts			0.49^b^
Yes	0 (0)	2 (3.3)	
No	60 (100)	59 (96.7)	
Level of interleukin 1			0.120^a^
Mean ± SD	23 ± 28.11	19.03 ± 19.43	
Level of interleukin 6			0.124^a^
Mean ± SD			
	180.25 ± 204.81	143.77 ± 170.12	

a Independent *t*-test or Mann-Whitney U.

b Chi-square.

The results showed that the mean and standard deviation of the patients’ temperature on the first, third, and fifth days was 37.94 ± 0.88, 37.57 ± 0.66, and 37.28 ± 0.48, respectively which showed a significant difference between the days based on Friedman test (*p* < 0.0001). The mean and standard deviation of the percentage of oxygen saturation in patients on the first, third, and fifth days was 92.36 ± 2.73, 92.76 ± 3.40, and 93.08 ± 3.77, respectively which showed a significant difference between the days based on Friedman test (*p* < 0.0001). According to the results of Cochran’s Q test, the rate of shortness of breath on the first, third, and fifth days was 90.1%, 54.5%, and 32.2%, respectively which showed a downward trend and a statistically significant difference between the 3 days (*p* < 0.0001). The rate of coughing on the first, third, and fifth days was 76.9%, 67.8%, and 50.4%, respectively which showed a downward trend and a statistically significant difference between the 3 days (*p* < 0.0001). As far as weakness and lethargy were concerned, their rate was 97.5%, 97.5%, and 96.7% on the first, third, and fifth days, respectively which showed a downward trend but no statistically significant difference (*p* = 0.368) (Table 3).

**Table 3:** Variation in physical problems of COVID-19 patients during the study period.

Variable	Day 1	Day 3	Day 5	*p*
*Body temperature*				
Mean ± SD	37.94 ± 0.88	37.57 ± 0.66	37.28 ± 0.48	<0.0001^a^
*Percentage of blood oxygen saturation*				
Mean ± SD	92.36 ± 2.73	92.76 ± 3.40	93.08 ± 3.77	<0.0001^a^
*Shortness of breath (%)*				
Yes	109 (90.2)	66 (54.5)	39 (32.2)	<0.0001^b^
No	12 (9.8)	55 (45.5)	82 (67.8)	
*Coughing (%)*				
Yes	93 (76.9)	82 (67.8)	61 (50.4)	<0.0001^b^
No	28 (23.1)	39 (32.2)	60 (49.6)	
*Weakness and lethargy (%)*				
Yes	118 (97.5)	118 (97.5)	117 (96.7)	0.368^b^
No	3 (2.5)	3 (2.5)	4 (3.3)	

a Friedman test.

b Cochran’s Q test.

The results showed that the mean and standard deviation of interleukin 1 level in the studied patients was 21.007 ± 12.24. Pearson’s correlation test showed that there was a direct and statistically significant correlation between body temperature on the first day and interleukin 1 level (*r* = 0.186, *p* = 0.041). However, no such correlation was observed between body temperature on the third (*r* = 0.130, *p* = 155, *p* = 0.0) and fifth days (*r* = 0.070, *p* = 0.447) and interleukin 1 level. A negative and statistically significant correlation was observed between blood oxygen saturation level and level of interleukin-1 on the first (*r* = −0.214, *p* = 0.018), third (*r* = −0.285, *p* = 0.002) and fifth days (*r* = −0.236, *p* = 0.009). According to the results of the statistical test, shortness of breath and coughing on the third and fifth days were significantly correlated with the level of interleukin 1 (*p* < 0.05) (Table 4).

**Table 4:** The relationship between physical problems (body temperature, blood oxygen saturation level, shortness of breath, coughing, weakness, and lethargy) and interleukin 1 level in patients with COVID-19.

Variable	Day 1	Day 3	Day 5
Body temperature	*r* = 0.186	*r* = 0.130	*r* = 0.07
*p* ^a^	0.041	0.155	0.447
Blood oxygen saturation level	*r* = −0.214	*r* = −0.285	*r* = −0.236
*p* ^a^	0.018	0.002	0.009
*Shortness of breath: frequency (%)*			
Yes	25.17 ± 21.89	29.82 ± 27.53	21.81 ± 26.48
No	7.17 ± 12.91	10.40 ± 13.17	21.58 ± 18.40
*p* ^b^	0.298	<0.0001	0.027
*Coughing: frequency (%)*			
Yes	24.92 ± 21.94	26.10 ± 27.96	16.07 ± 23.27
No	21.39 ± 17.90	18.96 ± 16.88	30.17 ± 18.70
*p* ^b^	0.088	0.034	0.004
*Weakness and lethargy: frequency (%)*			
Yes	24.34 ± 21.02	24.34 ± 21.02	24.43 ± 21.10
No	16.24 ± 20.50	16.24 ± 20.50	14.03 ± 18.20
*p* ^b^	0.940	0.944	0.925

a Pearson correlation test.

b Independent *t*-test or Mann-Whitney U.

According to the results of the Pearson correlation coefficient test, a direct and statistically significant correlation was observed between body temperature on the first (*r* = 0.339, *p* < 0.0001), third (*r* = 0.336, *p* < 0.0001) and fifth days (*r* = 0.244, *p* < 0.007, *p* = 0) and interleukin 6 level. Also, a negative and statistically significant correlation was observed between the percentage of blood oxygen saturation level on the first (*r* = −0.472, *p* < 0.0001), third (*r* = −0.598, *p* < 0.0001) and fifth days (*r* = −0.511, *p* < 0.0001) and interleukin 6 level. Coughing on the third and fifth days had a statistically significant relationship with the level of interleukin 6 (*p* < 0.05). According to the results of the statistical test, coughing on the third and fifth days had a statistically significant correlation with the level of interleukin 6 (*p* < 0.05) (Table 5).

**Table 5:** The correlation between physical problems (body temperature, blood oxygen saturation level, shortness of breath, coughing, weakness, and lethargy) and interleukin-6 level in COVID-19 patients.

Variable	Day 1	Day 3	Day 5
Body temperature	0.339	0.336	0.244
*p* ^a^	<0.0001	<0.0001	0.007
Blood oxygen saturation level	−0.472	−0.598	−0.511
*p* ^a^	<0.0001	<0.0001	<0.0001
*Shortness of breath: frequency (%)*			
Yes	173.91 ± 192.24	233.51 ± 210.04	260.66 ± 239.86
No	52.36 ± 95.68	75.87 ± 108.75	114.87 ± 136.25
*p* ^b^	0.002	<0.0001	<0.0001
*Coughing: frequency (%)*			
Yes	175.87 ± 196.21	190.45 ± 220.99	229.16 ± 216.32
No	115.33 ± 152.87	101.74 ± 136.33	93.44 ± 122.60
*p* ^b^	0.231	0.017	<0.0001
*Weakness and lethargy: frequency (%)*			
Yes	162.33 ± 189.30	162.33 ± 189.30	163.66 ± 189.55
No	143.46 ± 170.41	143.46 ± 170.41	109.17 ± 155.12
*p* ^b^	0.931	0.931	0.369

a Pearson correlation test.

b Independent *t*-test or Mann-Whitney U.

The results of the generalized estimating equations showed that with the increase in interleukin-1 (after adjusting for age and gender), the percentage of oxygen saturation decreases significantly (*p* = 0.014). Also, with the increase in interleukin-1 (after adjusting for age and gender), body temperature, shortness of breath, coughing, and weakness increase non-significantly (*p* < 0.05). The results of the generalized estimating equations showed that with the increase in interleukin 6 (after adjustment of age and gender), the percentage of oxygen saturation decreases significantly (*p* < 0.0001). The results also showed that with the increase in interleukin 6 (after adjusting for age and gender), temperature (*p* < 0.0001), shortness of breath (*p* < 0.0001) and coughing increase significantly (*p* = 0.023). Also, with the increase in interleukin-1 (with adjustment of age and gender), the rate of weakness and lethargy increases non-significantly (*p* = 0.971) (Table 6).

**Table 6:** Investigating the relationship between the severity of physical problems (i.e., body temperature, blood oxygen saturation percentage, shortness of breath, coughing, and weakness and lethargy) and the serum level of interleukin 1 and 6 in patients with COVID-19.

Outcome	Independent variable	Estimated coefficient (CI: 95%)	*p*
Body temperature	Interleukin 1	0.004 (−0.003, 0.01)	0.271
Percentage of blood oxygen saturation	Interleukin 1	−0.031 (−0.056, −0.006)	0.014
Shortness of breath	Interleukin 1	0.02 (−0.002, 0.04)	0.071
Coughing	Interleukin 1	0.009 (−0.01, 0.03)	0.399
Weakness and lethargy	Interleukin 1	0.002 (−0.02, 0.02)	0.876
Body temperature	Interleukin 6	0.001 (0.000, 0.002)	<0.0001
Percentage of blood oxygen saturation	Interleukin 6	−0.009 (−0.011, −0.006)	<0.0001
Shortness of breath	Interleukin 6	0.004 (0.002, 0.006)	<0.0001
Coughing	Interleukin 6	0.003 (0.00, 0.006)	0.023
Weakness and lethargy	Interleukin 6	0.000 (−0.007, 0.007)	0.971

The results of the generalized estimation equations showed that with the increase in interleukin-1 (after adjusting for age and gender), psychological problems (depression, anxiety, and stress) increase. Meanwhile, the increase in anxiety is statistically significant (*p* = 0.043). The results showed that with the increase in interleukin-6 (after adjusting for age and gender), psychological problems (depression, anxiety, and stress) increase significantly (*p* < 0.05) (Table 7).

**Table 7:** The relationship between the severity of psychological problems (depression, anxiety and stress) and the serum level of interleukin 6 in patients with COVID-19.

Mental problem	Independent variable	Estimated coefficient (95% confidence level)	*p*
Depression	IL-6	0.004 (0.001, 0.006)	0.007
Anxiety	IL-6	0.006 (0.003, 0.01)	<0.0001
Stress	IL-6	0.005 (0.001, 0.008)	0.006
Depression	IL-1	0.002 (−0.01, 0.02)	0.870
Anxiety	IL-1	0.02 (0.001, 0.05)	0.043
Stress	IL-1	0.02 (−0.004, 0.04)	0.101

## Discussion

The results of the present study showed that there was a direct and statistically significant correlation between body temperature on the first day and interleukin 1 level. A negative and statistically significant correlation was also observed between blood oxygen saturation level and interleukin-1 level on the first, third and fifth days. Shortness of breath and coughing on the third and fifth days had a statistically significant correlation with the level of interleukin 1.

Previous studies have shown that release of inflammatory cytokines and the release of blood neutrophils and monocytes into the bronchi will cause damage to the lung tissue and subsequently lead to destruction of alveolar ventilation (14). Based on these results and those of the present study, it can be argued that an increase in interleukin level is associated with reduced levels of blood oxygen saturation. Also, this alveolar destruction is followed by shortness of breath and coughing, which is in line with the results of the present study. As far as body temperature is concerned, it can be said that an increase in inflammatory mediators can lead to a further increase in the activity of the immune system.

The results of the current study also showed a direct and statistically significant correlation between body temperature on the first, third and fifth days and interleukin 6 level. A negative and statistically significant correlation was observed between blood oxygen saturation level on the first, third, and fifth days and interleukin 6 level. Coughing on the third and fifth days had a statistically significant relationship with the level of interleukin 6.

In a study aimed at determining the level of interleukin 6 in patients with COVID-19, Coomes et al. (30) found that the level of interleukin 6 in patients with COVID-19 significantly increased and was associated with adverse clinical outcomes, and that inhibition of interleukin 6 is a possible therapeutic target for managing dysregulated responses in patients with COVID-19. In COVID-19, interleukin-6 level is elevated (15, 16), and due to its mediating role in the lung and destruction of pulmonary alveoli (14), side effects such as drop in blood oxygen saturation level and coughing are expected, which is consistent with the results of the present study.

Our findings also showed no direct and non-significant statistical correlation between depression and stress and the serum level of interleukin 1. However, a significant correlation was observed between anxiety and the serum level of interleukin 1. Mazza et al. (6) studied anxiety and depression in COVID-19 survivors and the role of inflammatory and clinical predictors in this disease. They reported that the levels of peripheral lymphocytes, neutrophils, and platelets are associated with depression and anxiety scores in the follow-up stage. Although their study was on other factors related to inflammation, it seems that mental problems including anxiety can affect the production of inflammatory mediators by affecting the immune system. Additional studies on other viral diseases are recommended to provide more solid evidence for such conclusions.

According to the results of the present study, depression, anxiety and stress had a direct and statistically significant correlation with the serum level of interleukin 6. In a review study, Sepehrinezhad et al. (22) reported that chronic stress by inducing the production of pro-inflammatory cytokines such as interleukin-6 can lead to an increase in various chronic diseases, which confirms the results of the present study. O’Donovan’s et al. (31) study showed significantly higher levels of interleukin-6 in participants with clinical anxiety compared to non-anxious participants. Although their study population is different from that of the present study, both studies came up with similar results. Also, another study showed that the level of interleukin 6 in women having positive social relationships and a purpose for life is lower compared with other women (19), suggesting that people experiencing anxiety, depression and anxiety have a higher level of interleukins in their bodies. This has important implications for nurses and health officials, because nurses, as the main caregivers of patients, especially during the COVID-19 pandemic, play an important role in reducing mental stress caused by this pandemic. In fact, nurses’ care measures to reduce these symptoms can play an effective role in reducing inflammatory mediators. Of course, additional studies in this regard can help to provide clinical evidence for this.

A limitation of this study is the self-reporting of symptoms that might bias the analysis, since participants with ongoing symptoms and higher subjective symptom load may be select. Second, we used IL-6 and IL-1 level as a biomarker of inflammatory cytokines. Future studies should consider other inflammatory cytokines such as C-reactive protein, L-7, IL-8, and granulocyte colony stimulating factor and TNF-α. Finally, it was not possible to examine and eliminate other confounding factors related to mental problems in these patients.

## Conclusion

Given the relationship between levels of interleukin-1 and -6 and most physical and mental problems, it seems that the level of these inflammatory biomarkers can be used to estimate the severity of physical and psychological symptoms in COVID-19 patients. Furthermore, researchers should develop a scoring system including IL-1 and IL-6 to assist clinicians in early recognition of patients at risk for developing severe disease.

## Data availability statement

The datasets presented in this article are not readily available because these data were used under license for the current study, and so are not publicly available. Data are, however available from the authors upon reasonable request. Requests to access the datasets should be directed to SM, shahrambaraz@ajums.ac.ir.

## Ethics statement

The studies involving humans were approved by Ahvaz Jundishapur University of Medical Sciences. The studies were conducted in accordance with the local legislation and institutional requirements. Written informed consent for participation in this study was provided by the participants’ legal guardians/next of kin.

## Author contributions

GB-B, SM, MR, FA, EM, BD, ZU, and GA-A: investigation, wrote the manuscript, and writing—review and editing. GB-B, SM, MR, FA, EM, and BD: conceptualization methodology and writing—review and editing. GB-B, SM, BD, EM, MR, and ZU: writing—review and editing. All authors contributed to the article and approved the submitted version.

## Funding

This study was drawn from a research project (U-00046) sponsored by Deputy of Research and Technology of AJUMS. The payment was spent on the design and implementation of the study.

## Conflict of interest

The authors declare that the research was conducted in the absence of any commercial or financial relationships that could be construed as a potential conflict of interest.

## Publisher’s note

All claims expressed in this article are solely those of the authors and do not necessarily represent those of their affiliated organizations, or those of the publisher, the editors and the reviewers. Any product that may be evaluated in this article, or claim that may be made by its manufacturer, is not guaranteed or endorsed by the publisher.
